# Supplementary material for: Trans-eQTL mapping prioritises USP18 as a negative regulator of interferon response at a lupus risk locus
Source: Nat Commun. 2025 Oct 2;16:8795. doi: 10.1038/s41467-025-63856-7 (PMC12491431; doi:10.1038/s41467-025-63856-7)
Supplement: Supplementary file 1 — Supplementary Information [file 41467_2025_63856_MOESM1_ESM.pdf]

Trans-eQTL mapping prioritises *USP18* as a negative regulator of interferon response at a lupus risk locus

Supplementary Notes

Supplementary Note 1: MYBL2 regulates the expression of many cell cycle genes
Supplementary Note 2: Estimating the sample size required to experimentally validate the effect of USP18 missense variant on target gene expression

Supplementary Tables

Supplementary Table 1: Overview of the LCL eQTL discovery cohorts.

Supplementary Figures

Supplementary Fig. 1: Overview of *trans*-eQTL results at the relaxed $p < 5\times10^{-8}$ threshold.
Supplementary Fig. 2: Forest plots of cohort-specific effect size for the four *trans*-eQTL loci that replicated in the MAGE cohort.
Supplementary Fig. 3: Colocalisation between the SLE GWAS signal from the FinnGen + UKBB + MVP meta-analysis and the *USP18 trans*-eQTL locus detected in our analysis.
Supplementary Fig. 4: Original regional association plot for the *USP18* SLE GWAS locus from Yin *et al.* 2020 study.
Supplementary Fig. 5: Category III: other interferon alpha/beta signalling pathway genes that do not belong to categories I or II (shown in Fig. 3)
Supplementary Fig. 6: Fine-mapped splicing QTL (sQTL) in the *OAS1* gene.
Supplementary Fig. 7: Expression level of *USP18* in resting and stimulated B-cell subset of peripheral blood mononuclear cells (PBMCs).
Supplementary Fig. 8: Forest plots of cohort-specific effect size for the remaining *trans*-eQTL loci that either did not replicate in the MAGE cohort or corresponded to likely cross-mappability artefacts (*SENP7*, *ZNF781* and *ZBTB10* loci).
Supplementary Fig. 9: MYBL2 regulates the expression of many cell cycle genes.
Supplementary Fig. 10: Overlap between *trans*-eQTL target genes and MYBL2 ChIP-seq peaks.

Supplementary References

Supplementary Note 1

MYBL2 regulates the expression of many cell cycle genes

At the *MYBL2* locus, the lead variant chr20_43721344_C_T was associated with the expression of 151 target genes at FDR 5% (Figure S9). The target genes were strongly enriched for the Gene Ontology mitotic cell cycle term (GO:0000278, $p=2.607\times10^{-51}$) and the Reactome mitotic cell cycle pathway (R-HSA-69278, $p=1.216\times10^{-35}$). Interestingly, 125/151 genes (82%) had lower expression in carriers of the alternative T allele (Figure S9). The T allele of chr20_43721344_C_T was also strongly associated ($p = 8.83\times10^{-218}$) with the decreased expression of the *MYBL2* transcription factor gene in *cis (*Figure S9). Since both the MYBL2 transcription factor located in *cis* and majority of the *trans*-genes had lower expression in the carriers of the T allele, we hypothesised that MYBL2 might directly regulate these target genes. To test this, we download ChIP-seq data for the MYBL2 transcription factor in the human K562 myelogenous leukemia cell line from the ENCODE project (ENCSR162IEM). We then asked how many of the up- and downregulated genes had a MYBL2 ChIP-seq peak within +/- 2kb from the annotated promoter of the gene. We found that 99/125 (78.4%) downregulated genes had a MYBL2 peak in their promoter region (Figure S10). In contrast, only 1/26 upregulated genes had a MYBL2 peak in their promoter region. As a negative control, we looked at the 404 5% FDR target genes of the *SP140* locus (Table S2) and found that only 23/404 (5.6%) of the target genes had a MYBL2 peak in their promoter region (Figure S10).

To further understand which cell cycle stage these *MYBL2* target genes might be involved in, we obtained the list of genes specific to G2M and S phases of the cell cycle from Tirosh et al. 2015[1] using Seurat R package[2]. We found that 33/125 genes downregulated by the *trans*-eQTL variant were markers of the G2M phase which was significantly more than expected ($p = 1.23\times10^{-53}$). In contrast, only 1/125 downregulated genes overlapped with markers of the S phase ($p = 0.36$). Of note, 2/26 upregulated genes overlapped S-phase markers ($p = 0.004$) and none of the upregulated genes overlapped G2M-phase markers.

Altogether, this evidence strongly suggests that MYBL2 directly regulates the expression of G2M genes in *trans* by binding to their promoter sequences and is directly involved in the regulation of the expression of these target genes.

Supplementary Note 2

Estimating the sample size required to experimentally validate the effect of *USP18* missense variant on target gene expression

To see if it would be possible to experimentally validate the effect of the rs3180408 *USP18* missense variant on *trans*-eQTL target gene expression, we turned to power calculations. The largest *trans*-eQTL effect in our discovery cohort was detected for the *HERC5* gene (beta = 0.15). Given that the MAF of the rs3180408 was 0.346, this means that the rs3180408 variant explained ~1% of the variance in the expression of the *HERC5* gene. However, the effect size estimate in the discovery cohort is likely to be overestimated due to the winner's curse[3]. In the MAGE replication cohort (n = 682), the effect size of the rs3180408 variant was beta = 0.094, which corresponds to ~0.4% of the variance explained in the *HERC5* gene expression. This is a very small effect.

To validate our simulation approach, we first sought to estimate the sample size required in a standard *trans*-eQTL mapping setting where the variant has a MAF = 0.346 and beta = 0.1. Averaging the results from 1000 simulations, we found that we need approximately 800 samples to have ~80% power to detect a significant effect ($p < 0.05$). This is consistent with our empirical replication results on the MAGE cohort (n = 682), where we had beta = 0.094 and $p = 0.01$.

In an ideal experimental validation scenario, we would be able to engineer this variant into a lymphoblastoid cell line in an homozygous state (i.e. change both alleles). This is expected to reduce the required sample size, because now the expected effect size is twice as large (we only compare homozygous individuals) and we can ensure that both homozygotes are present at 50% frequency. This was confirmed in simulations where we found that we would need ~200 samples to have 80% power to detect the effect and ~300 samples to have 95% power to detect the effect.

One way to design such an experiment would be to start with 10 independent lymphoblastoid cell lines homozygous for rs3180408-C reference allele, perform genome editing to change both copies of the C allele to T allele, isolate successfully edited clones and then measure *HERC5* expression in each clone and matched control cell line 10-15 times (200-300 samples total). Finally, at this scale, various technical and batch effects are likely to have a much larger effect than the (very small) genetic effect we are trying to ascertain, further complicating interpretation. Thus, even if we were able to perform genome editing in LCLs or primary B cells (which is far from obvious), the expected effect size of the missense variant is too small to be detectable in this setting.

Supplementary Table 1. **Overview of the LCL eQTL discovery cohorts.** The cohorts included in the analysis used a mixture of RNA-seq and microarray technologies and three cohorts (TwinsUK, MRCE and MRCA) contained related samples. In the sample size column, the numbers in parentheses show the number of female (F) and male (M) samples in each cohort.

Cohort	Sample size	Expression data	Genotype data	Relatedness
ALSPAC[4–6]	876 (463 F, 413 M)	microarray	imputed (1000G 30x on GRCh38)	unrelated
TwinsUK[7]	735 (F)	RNA-seq	imputed (1000G 30x on GRCh38)	twins
CoLaus[8,9]	553 (293 F, 260 M)	RNA-seq	imputed (TOPMed)	unrelated
GEUVADIS[10]	358 (187 F, 171 M)	RNA-seq	WGS (1000G 30x on GRCh38)	unrelated
Liang_2013 (MRCE)[11]	484 (243 F, 241 M)	microarray	imputed (1000G 30x on GRCh38)	siblings
Liang_2013 (MRCA)[11]	325 (144 F, 181 M)	microarray	imputed (1000G 30x on GRCh38)	siblings
GENCORD[12]	187 (88 F, 99 M)	RNA-seq	imputed (1000G 30x on GRCh38)	unrelated
GTEx[13]	113 (43 F, 70 M)	RNA-seq	WGS (GRCh38)	unrelated
CAP[14]	100 (42 F, 58 M)	RNA-seq	imputed (1000G 30x on GRCh38)	unrelated
MAGE[15] (replication)	682 (357 F, 325 M)	RNA-seq	WGS (GRCh38)	unrelated, diverse ancestries

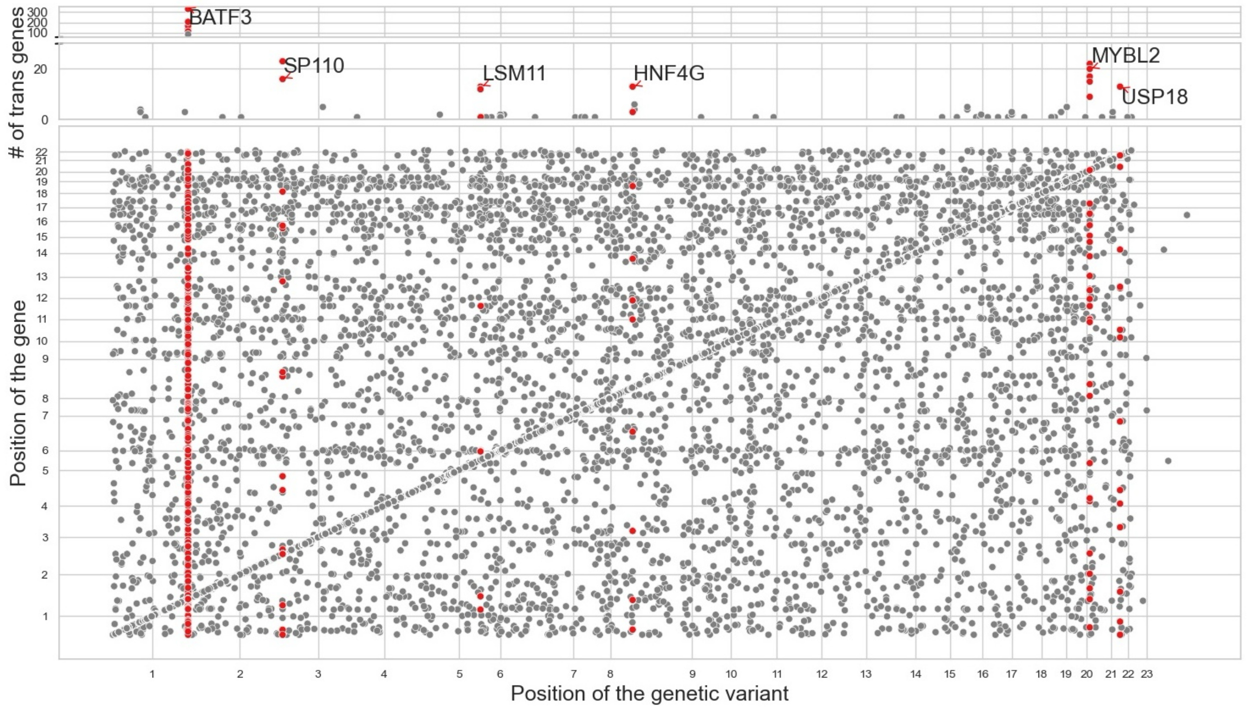

Supplementary Fig. 1. **Overview of *trans*-eQTL results at the relaxed p < 5×10⁻⁸ threshold**. The upper scatter plot shows the number of *trans* associations detected at each *trans*-eQTL locus with p-values < 5×10^{-8}. Six largest *trans*-eQTL loci have been labelled with the name of the closest *cis* gene. The lower scatter plot shows all significant loci for each tested gene at the $p < 5\times10^{-8}$ threshold. *Cis* associations are located on the diagonal while putative *trans* associations are located off diagonal.

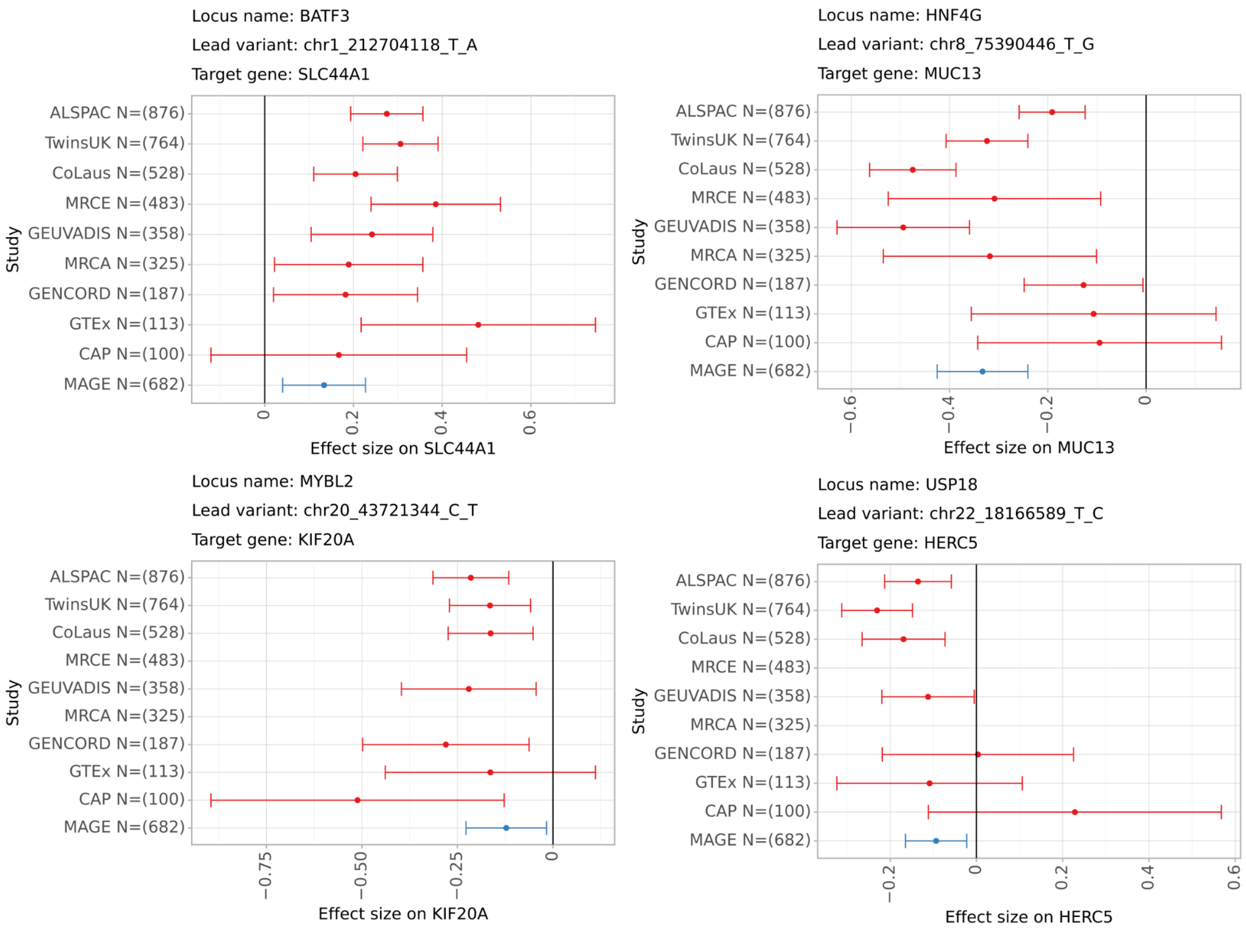

Supplementary Fig. 2. **Forest plots of cohort-specific effect size for the four *trans*-eQTL loci that replicated in the MAGE cohort.** The points represent the *trans*-eQTL effect size estimates from regenie and the error bars represent 95% confidence intervals. The four panels correspond to the *BATF3*, *HNF4G*, *MYBL2* and *USP18* loci, their respective lead variants and lead target genes.

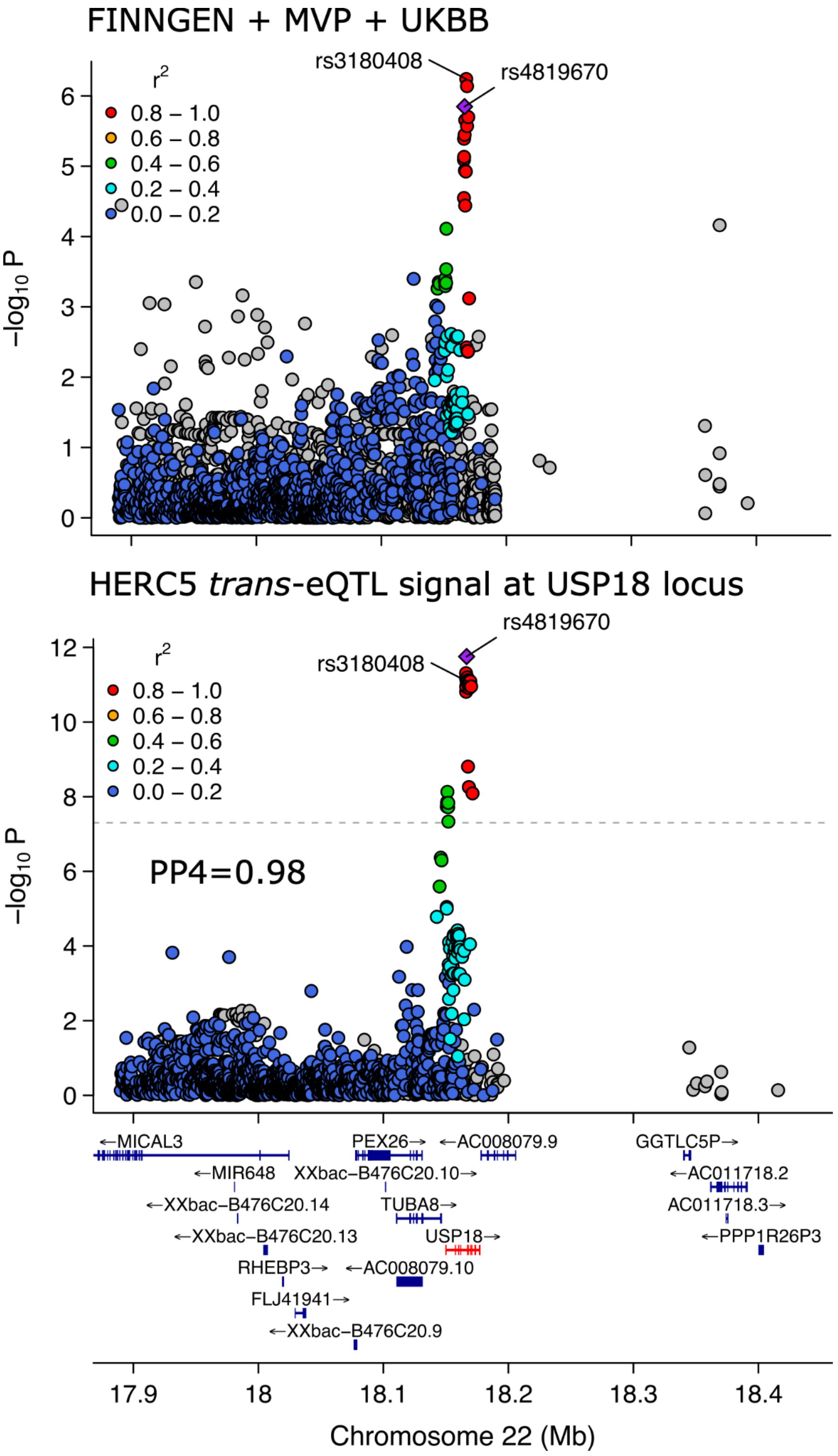

Supplementary Fig. 3. Colocalisation between the SLE GWAS signal from the FinnGen + UKBB + MVP meta-analysis and the *USP18 trans*-eQTL locus detected in our analysis.

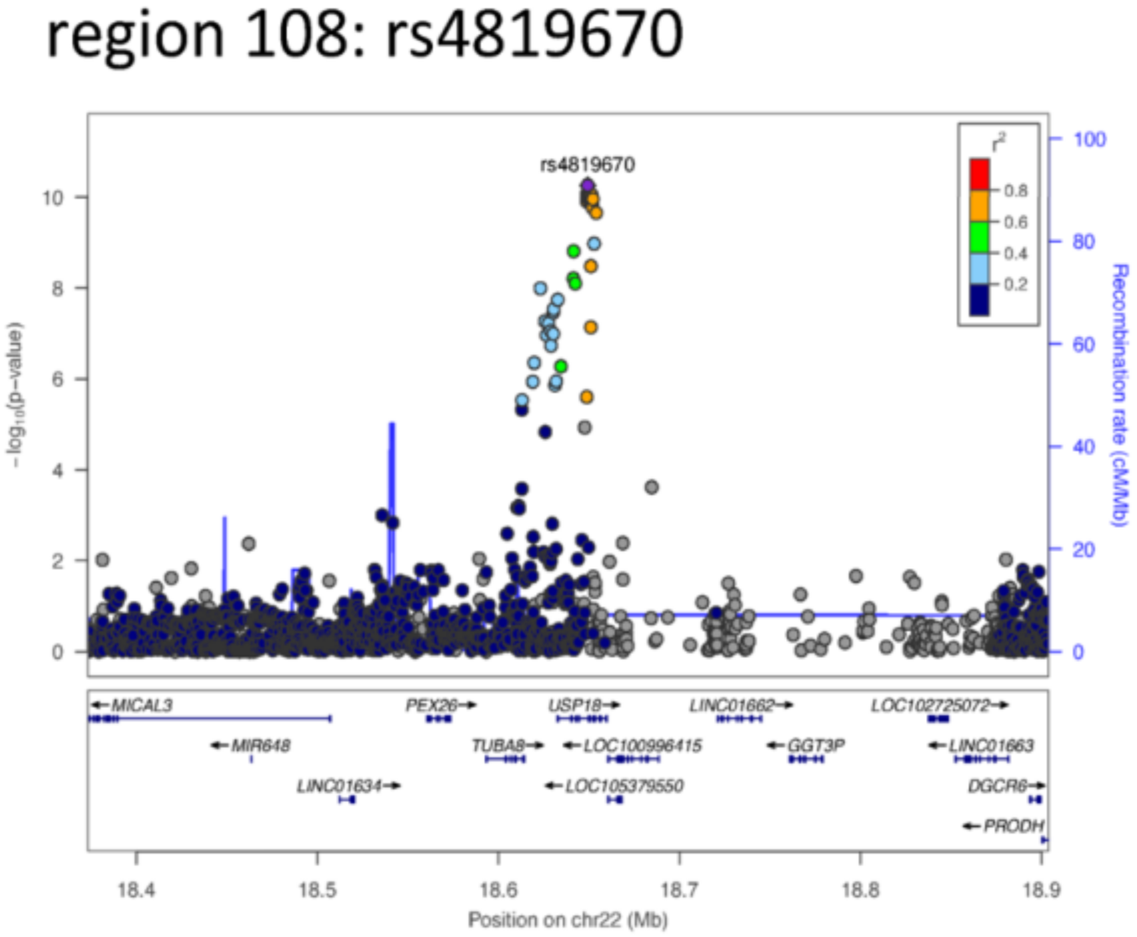

Supplementary Fig. 4. Original regional association plot for the *USP18* SLE GWAS locus from Yin *et al.* 2020 study.

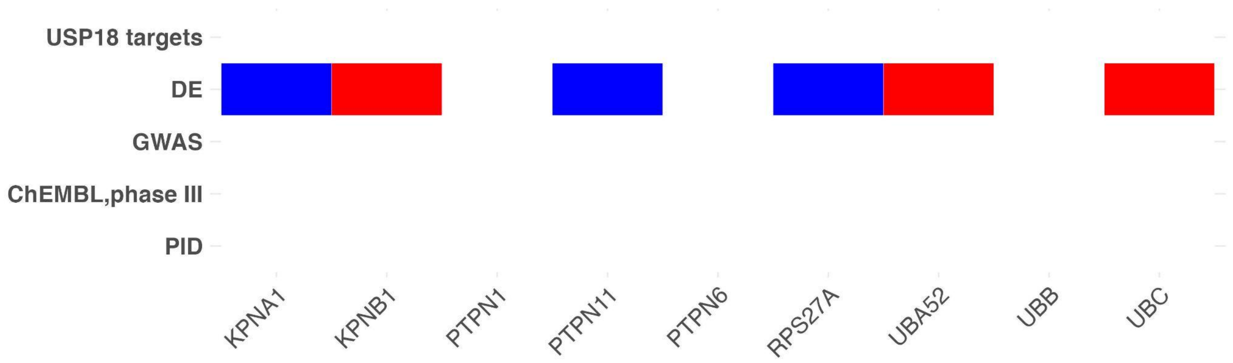

Supplementary Fig. 5. Category III: other interferon alpha/beta signalling pathway genes that do not belong to categories I or II (shown in Fig. 3). The increased gene expression is marked in red, while reduced gene expression is marked in blue. The visualisation illustrates the effect on USP18 targets in relation to the risk allele. DE - differential gene expression in SLE cases *versus* controls[16]; GWAS - GWAS hits for SLE[17], ChEMBL, phase III - SLE phase III clinical trials from ChEMBL[18], PID - genes causing primary immunodeficiency from Genomics England.

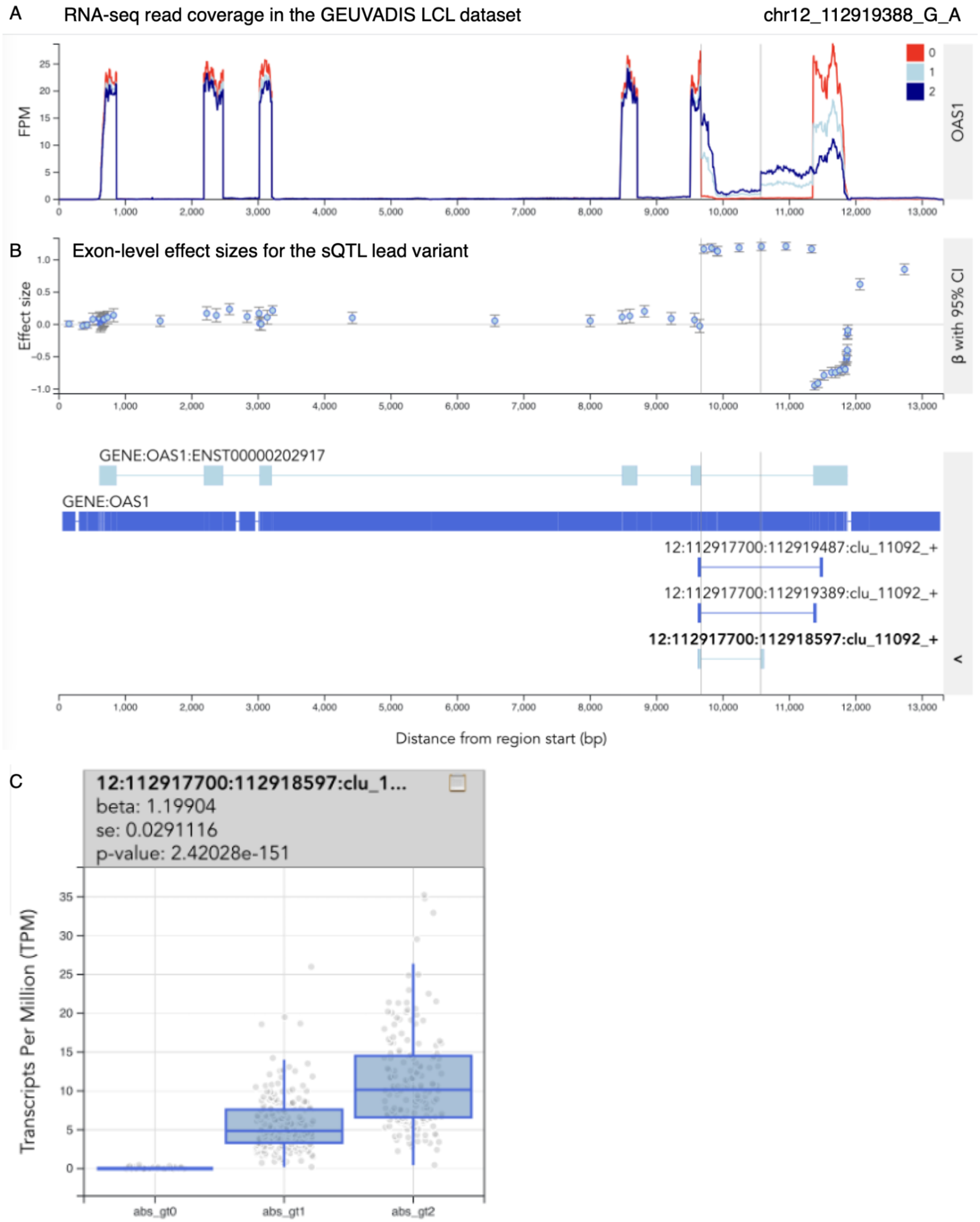

Supplementary Fig. 6. Fine-mapped splicing QTL (sQTL) in the *OAS1* gene. (**A**) RNA-seq read coverage of the *OAS1* gene in the GEUVADIS LCL dataset, stratified by the genotype of the fine-mapped sQTL variant chr12_112919388_G_A (posterior inclusion probability = 1). (**B**) Exon-level effect sizes for the sQTL lead variant. (**C**) Boxplot of the absolute expression of the short last intron of the *OAS1* gene (highlighted on panel A) stratified by the genotype of the lead sQTL variant. Interactive version of the plot can be viewed here.

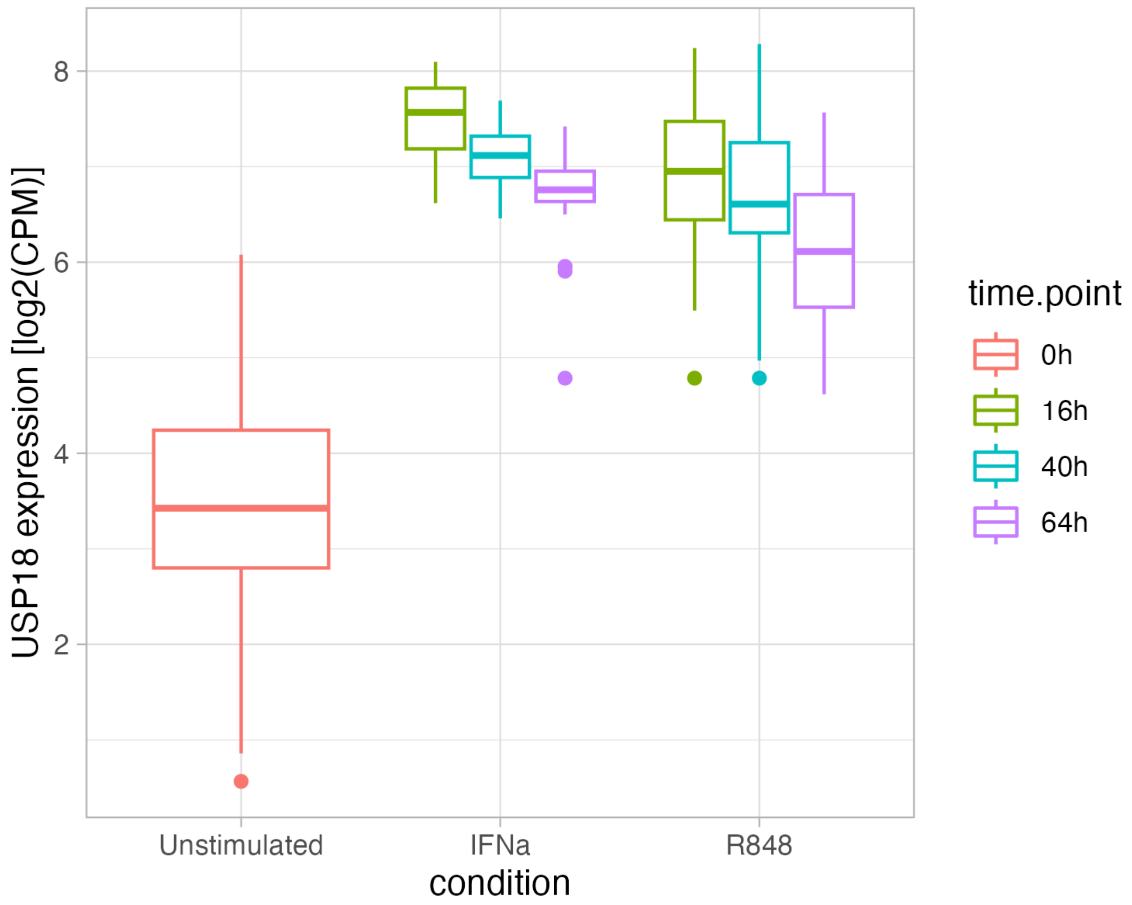

Supplementary Fig. 7. **Expression level of *USP18* in resting and stimulated B-cell subset of peripheral blood mononuclear cells (PBMCs)**. PBMCs were isolated from healthy donors and stimulated with interferon-alpha (IFNa) or R848 for 16, 40 and 64 hours.

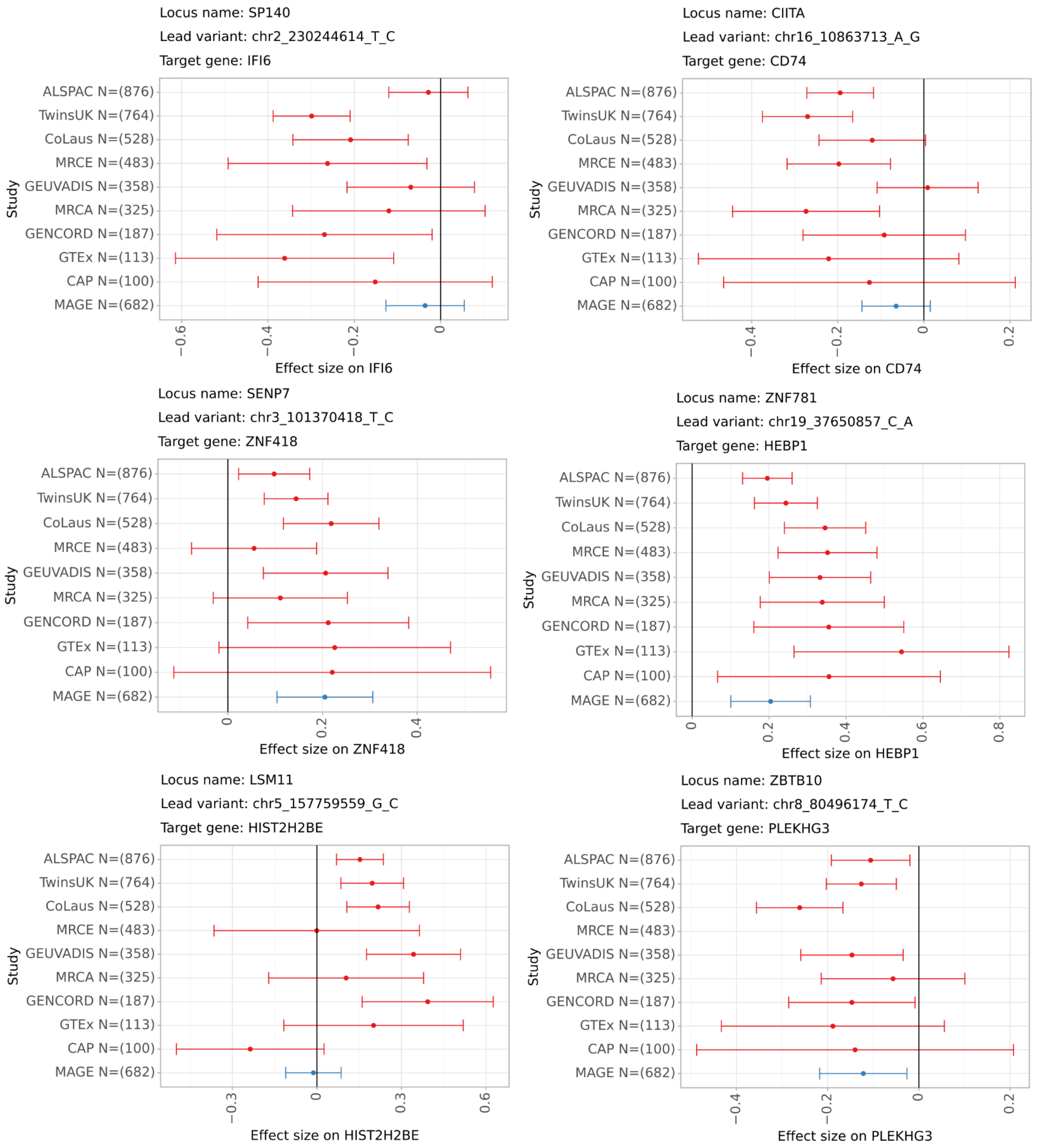

Supplementary Fig. 8. Forest plots of cohort-specific effect size for the remaining *trans*-eQTL loci that either did not replicate in the MAGE cohort or corresponded to likely cross-mappability artefacts (*SENP7*, *ZNF781* and *ZBTB10* loci). The points represent the *trans*-eQTL effect size estimates from regenie and the error bars represent 95% confidence intervals.

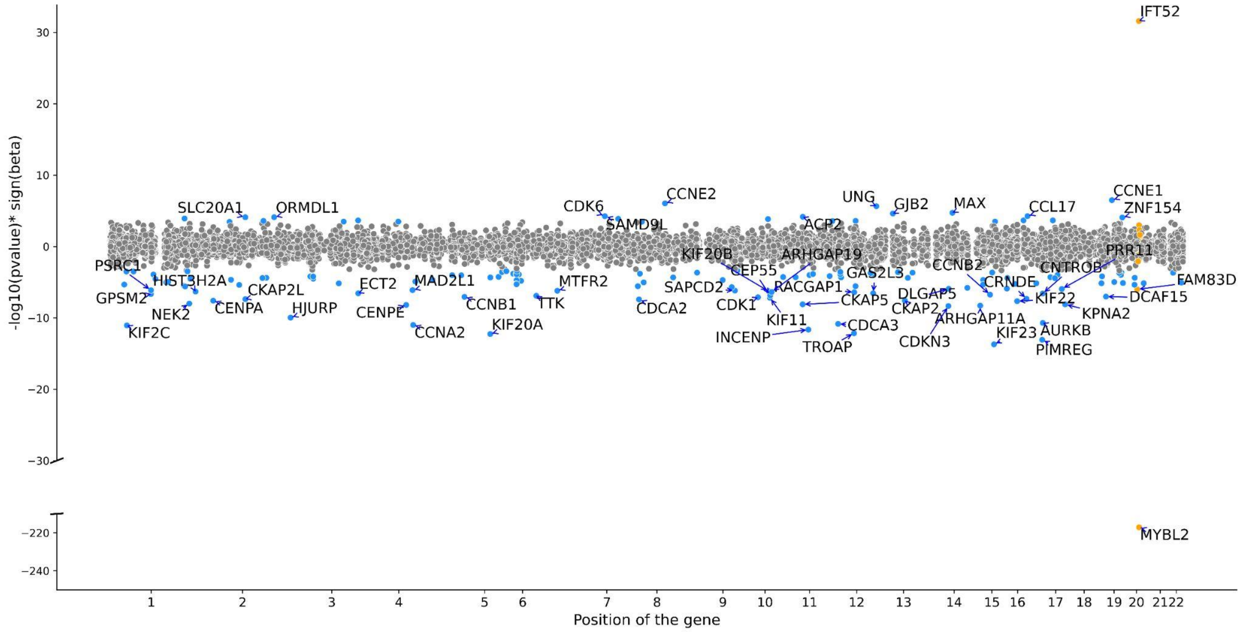

Supplementary Fig. 9. **MYBL2 regulates the expression of many cell cycle genes.** The scatter plot shows all genes associated with the *MYBL2 trans*-eQTL lead variant (chr20_43721344_C_T). Light blue points show significantly associated genes (variant-level Benjamini-Hochberg FDR 5%)

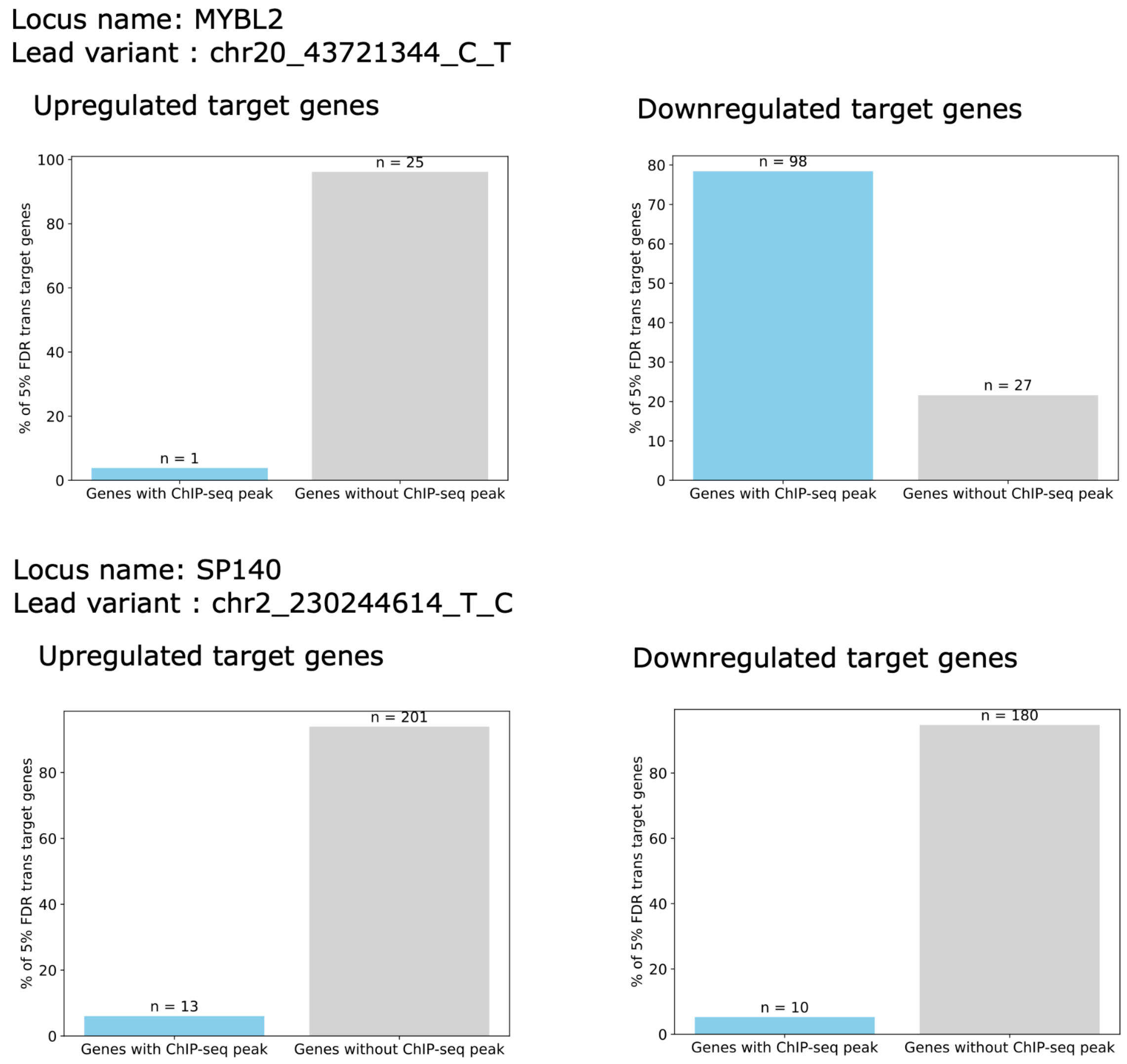

Supplementary Fig. 10. Overlap between *trans*-eQTL target genes and MYBL2 ChIP-seq peaks. The top panel shows the proportion of the *MYBL2 trans*-eQTL target genes upregulated (left) or downregulated (right) by the effect allele that contain a MYBL2 ChIP-seq peak within +/- 2kb from the annotated promoter. The bottom panel shows the proportion of the *SP140 trans*-eQTL target genes upregulated (left) or downregulated (right) by the effect allele that contain a MYBL2 ChIP-seq peak within +/- 2kb from the annotated promoter. Only genes downregulated by the MYBL2 effect allele show a sizable overlap with MYBL2 ChIP-seq peaks.

Supplementary References

1. Kowalczyk, M. S. *et al.* Single-cell RNA-seq reveals changes in cell cycle and differentiation programs upon aging of hematopoietic stem cells. *Genome Res.* **25**, 1860–1872 (2015).
2. Hao, Y. *et al.* Dictionary learning for integrative, multimodal and scalable single-cell analysis. *Nat. Biotechnol.* **42**, 293–304 (2023).
3. Huang, Q. Q., Ritchie, S. C., Brozynska, M. & Inouye, M. Power, false discovery rate and Winner's Curse in eQTL studies. *Nucleic Acids Res.* **46**, e133 (2018).
4. Boyd, A. *et al.* Cohort Profile: the 'children of the 90s'--the index offspring of the Avon Longitudinal Study of Parents and Children. *Int. J. Epidemiol.* **42**, 111–127 (2013).
5. Fraser, A. *et al.* Cohort Profile: the Avon Longitudinal Study of Parents and Children: ALSPAC mothers cohort. *Int. J. Epidemiol.* **42**, 97–110 (2013).
6. Bryois, J. *et al.* Cis and trans effects of human genomic variants on gene expression. *PLoS Genet.* **10**, e1004461 (2014).
7. Buil, A. *et al.* Gene-gene and gene-environment interactions detected by transcriptome sequence analysis in twins. *Nat. Genet.* **47**, 88–91 (2015).
8. Firmann, M. *et al.* The CoLaus study: a population-based study to investigate the epidemiology and genetic determinants of cardiovascular risk factors and metabolic syndrome. *BMC Cardiovasc. Disord.* **8**, 6 (2008).
9. Sönmez Flitman, R. *et al.* Untargeted Metabolome- and Transcriptome-Wide Association Study Suggests Causal Genes Modulating Metabolite Concentrations in Urine. *J. Proteome Res.* **20**, 5103–5114 (2021).
10. Lappalainen, T. *et al.* Transcriptome and genome sequencing uncovers functional variation in humans. *Nature* **501**, 506–511 (2013).
11. Liang, L. *et al.* A cross-platform analysis of 14,177 expression quantitative trait loci derived

from lymphoblastoid cell lines. *Genome Res.* **23**, 716–726 (2013).

12. Gutierrez-Arcelus, M. *et al.* Passive and active DNA methylation and the interplay with genetic variation in gene regulation. *Elife* **2**, e00523 (2013).
13. Consortium, T. G. *et al.* The GTEx Consortium atlas of genetic regulatory effects across human tissues. *Science* **369**, 1318–1330 (2020).
14. Theusch, E., Chen, Y.-D. I., Rotter, J. I., Krauss, R. M. & Medina, M. W. Genetic variants modulate gene expression statin response in human lymphoblastoid cell lines. *BMC Genomics* **21**, 555 (2020).
15. Taylor, D. J. *et al.* Sources of gene expression variation in a globally diverse cohort. *bioRxiv* 2023.11.04.565639 (2023) doi:10.1101/2023.11.04.565639.
16. Banchereau, R. *et al.* Personalized Immunomonitoring Uncovers Molecular Networks that Stratify Lupus Patients. *Cell* **165**, 551–565 (2016).
17. Yin, X. *et al.* Meta-analysis of 208370 East Asians identifies 113 susceptibility loci for systemic lupus erythematosus. *Ann. Rheum. Dis.* **80**, 632–640 (2021).
18. Zdrazil, B. *et al.* The ChEMBL Database in 2023: a drug discovery platform spanning multiple bioactivity data types and time periods. *Nucleic Acids Res.* **52**, D1180–D1192 (2024).
