# Supplementary material for: Trans-eQTL mapping prioritises USP18 as a negative regulator of interferon response at a lupus risk locus
Source: Nat Commun. 2025 Oct 2;16:8795. doi: 10.1038/s41467-025-63856-7 (PMC12491431; doi:10.1038/s41467-025-63856-7)
Supplement: Supplementary file 2 — Description of Additional Supplementary Files [file 41467_2025_63856_MOESM2_ESM.pdf]

Description of Additional Supplementary Files

Supplementary Data 1: Replication of the prioritised trans-eQTL associations in the MAGE and eQTLGen Consortium datasets. The effect sizes, standard errors and two-sided p-values were calculated from an inverse-variance weighted meta-analysis of per-cohort linear model or linear mixed model regression effect size estimates.

Supplementary Data 2: Per-cohort regenie linear mixed model effect sizes and standard errors for the 50 USP18 trans-eQTL target genes.

Supplementary Data 3: Overlap of the UPS18 trans-eQTL target genes with Reactome interferon response pathway, primary immunodeficiency genes, SLE GWAS hits, known SLE drug targets, and differentially expressed genes in SLE cases versus controls.

Supplementary Data 4: Replication of the USP18 trans-eQTL target gene associations in the eQTLGen Consortium. The eQTLGen effect sizes, standard errors and two-sided p-values were calculated from an inverse-variance weighted meta-analysis of per-cohort linear regression effect size estimates.

Supplementary Data 5: Replication of the USP18 trans-eQTL target gene associations in naive B-cells from the OneK1K single-cell RNA-seq cohort. The effect sizes, standard errors and two-sided p-values were calculated from a standard linear regression implemented in the regenie software.

Supplementary Data 6: Examples of suspected cross-mappability artefacts in trans-eQTL analysis.
