# Supplementary material for: Trans-eQTL mapping prioritises USP18 as a negative regulator of interferon response at a lupus risk locus
Source: Nat Commun. 2025 Oct 2;16:8795. doi: 10.1038/s41467-025-63856-7 (PMC12491431; doi:10.1038/s41467-025-63856-7)
Supplement: Supplementary file 9 — Reporting Summary [file 41467_2025_63856_MOESM9_ESM.pdf]

nature portfolio

Corresponding author(s): Kaur Alasoo

Last updated by author(s): Jul 10, 2025

Reporting Summary

Nature Portfolio wishes to improve the reproducibility of the work that we publish. This form provides structure for consistency and transparency in reporting. For further information on Nature Portfolio policies, see our Editorial Policies and the Editorial Policy Checklist.

Statistics

For all statistical analyses, confirm that the following items are present in the figure legend, table legend, main text, or Methods section.

n/a	Confirmed	
☐	☒	The exact sample size (*n*) for each experimental group/condition, given as a discrete number and unit of measurement
☐	☒	A statement on whether measurements were taken from distinct samples or whether the same sample was measured repeatedly
☐	☒	The statistical test(s) used AND whether they are one- or two-sided<br>*Only common tests should be described solely by name; describe more complex techniques in the Methods section.*
☐	☒	A description of all covariates tested
☐	☒	A description of any assumptions or corrections, such as tests of normality and adjustment for multiple comparisons
☐	☒	A full description of the statistical parameters including central tendency (e.g. means) or other basic estimates (e.g. regression coefficient) AND variation (e.g. standard deviation) or associated estimates of uncertainty (e.g. confidence intervals)
☐	☒	For null hypothesis testing, the test statistic (e.g. *F*, *t*, *r*) with confidence intervals, effect sizes, degrees of freedom and *P* value noted<br>*Give P values as exact values whenever suitable.*
☐	☒	For Bayesian analysis, information on the choice of priors and Markov chain Monte Carlo settings
☐	☒	For hierarchical and complex designs, identification of the appropriate level for tests and full reporting of outcomes
☐	☒	Estimates of effect sizes (e.g. Cohen's *d*, Pearson's *r*), indicating how they were calculated

Our web collection on statistics for biologists contains articles on many of the points above.

Software and code

Policy information about availability of computer code

Data collection	No software was used for data collection.
Data analysis	For trans-eQTL analyis, we used CrossMap v0.4.1, Eagle v2.4.1 and Minimac4, bcftools v1.9.0, QCTOOL v2.2.0, Trim Galore v0.5.0, HISAT2 v2.2.1, featureCounts v1.6.4 and regenie v3.2.1. For single-cell data analysis we used cellSNP v1.2.1, Cell Ranger Multi pipeline v7.0.0, RStudio v4.3.1, Seurat v5.0.1, Azimuth v0.5.0, edgeR (v4.0.16), bedtools v2.19.0. The MetaLCL trans-eQTL analysis workflows are available from https://github.com/freimannk/regenie_analysis, the MetaLCL meta-analysis workflow is available from https://github.com/freimannk/regenie_metaanalyse. Additional documentation and code used to generate figures in the paper is available from https://github.com/AlasooLab/MetaLCL. The eQTL Catalogue genotype imputation, RNA-seq processing and data normalisation workflows are available from https://github.com/eQTL-Catalogue/genimpute, https://github.com/eQTL-Catalogue/rnaseq and https://github.com/eQTL-Catalogue/qcnorm. The eQTLGen genotype imputation, data quality control, data preparation and meta-analysis workflows are available from https://github.com/eQTLGen/eQTLGenImpute, https://github.com/eQTLGen/DataQC, https://github.com/eQTLGen/ConvertVcf2Hdf5, https://github.com/eQTLGen/PerCohortDataPreparations and https://github.com/eQTLGen/MetaAnalysis.

For manuscripts utilizing custom algorithms or software that are central to the research but not yet described in published literature, software must be made available to editors and reviewers. We strongly encourage code deposition in a community repository (e.g. GitHub). See the Nature Portfolio guidelines for submitting code & software for further information.

Data

Policy information about availability of data

All manuscripts must include a data availability statement. This statement should provide the following information, where applicable:

- Accession codes, unique identifiers, or web links for publicly available datasets
- A description of any restrictions on data availability
- For clinical datasets or third party data, please ensure that the statement adheres to our policy

The whole genome sequencing data for the GEUVADIS and MAGE studies was downloaded from the 1000 Genomes website (https://www.internationalgenome.org/data-portal/data-collection/30x-grch38). The GEUVADIS RNA-seq data was downloaded from the European Nucleotide Archive (ENA) under accession PRJEB3366 [https://www.ebi.ac.uk/ena/browser/view/PRJEB3366]. The MAGE RNA-seq data was downloaded from the ENA (accession PRJNA851328 [https://www.ebi.ac.uk/ena/browser/view/PRJNA851328]). The genotype and RNA-seq data from the GENCORD study was downloaded from European Genotype-phenotype Archive (EGA) under accessions EGAD00001000425 [https://www.ebi.ac.uk/ega/datasets/EGAD00001000425] and EGAD00001000428 [https://www.ebi.ac.uk/ega/datasets/EGAD00001000428]. The microarray gene expression data from the MRCA and MRCE studies was downloaded from ArrayExpress (accessions E-MTAB-1425 [https://www.ebi.ac.uk/biostudies/arrayexpress/studies/E-MTAB-1425] and E-MTAB-1428 [https://www.ebi.ac.uk/biostudies/arrayexpress/studies/E-MTAB-1428]) and the genotype data was downloaded from EGA (accession EGAS00000000137 [https://ega-archive.org/studies/EGAS00000000137]). The gene expression and genotype data from GTEx and CAP studies was downloaded from dbGaP (accessions phs000424.v8.p2 [https://www.ncbi.nlm.nih.gov/projects/gap/cgi-bin/study.cgi?study_id=phs000424.v8.p2] and phs000481.v3.p2 [https://www.ncbi.nlm.nih.gov/projects/gap/cgi-bin/study.cgi?study_id=phs000481.v3.p2]). The RNA-seq data from the TwinsUK study was downloaded from EGA (accession EGAD00001001086 [https://ega-archive.org/datasets/EGAD00001001086]) and genotype data was obtained from TwinsUK (https://twinsuk.ac.uk/researchers/access-data-and-samples/request-access/). The gene expression data from SLE cases and controls is available from GEO under accession code GSE65391 [https://www.ncbi.nlm.nih.gov/geo/query/acc.cgi?acc=GSE65391]. The informed consent obtained from ALSPAC participants does not allow the microarray and genotype data to be made freely available through any third party maintained public repository. However, data used for this study can be made available on request to the ALSPAC Executive. The ALSPAC data management plan describes in detail the policy regarding data sharing, which is through a system of managed open access. Full instructions for applying for data access can be found here: http://www.bristol.ac.uk/alspac/researchers/access/. The ALSPAC study website contains details of all the data that are available (http://www.bristol.ac.uk/alspac/researchers/our-data/). The RNA-seq and genotype data from the CoLaus cohort can be accessed by directly contacting the cohort (https://www.colaus-psycolaus.ch/professionals/how-to-collaborate/). The MetaLCL full trans-eQTL meta-analysis summary statistics are available from the eQTL Catalogue FTP server (https://www.ebi.ac.uk/eqtl/Data_access/) and additional documentation is available on the project website (https://github.com/AlasooLab/MetaLCL). Source data are provided with this paper.

Research involving human participants, their data, or biological material

Policy information about studies with human participants or human data. See also policy information about sex, gender (identity/presentation), and sexual orientation and race, ethnicity and racism.

Reporting on sex and gender	Due to limited sample size of our study and significant computational cost involved, we did not perform sex-stratified trans-eQTL analysis. Biological sex was included as a covariate in association testing.
Reporting on race, ethnicity, or other socially relevant groupings	The primary trans-eQTL analysis presented in this manuscript was performed using samples of primarily European genetic ancestries. Genetic ancestry was assessed on principal component analysis of the genotype data. No self-reported ancestry information was used. To avoid potential confounding by population stratification, we exclude the 87 YRI ancestry individuals from the GEUVADIS dataset and 34 diverse ancestry samples from the GTEx dataset.<br><br>Trans-eQTL replication was performed on the Multi-ancestry Analysis of Gene Expression dataset that contained individuals of diverse genetic ancestries from the 1000 Genomes Project. Genotype principal components were included as covariates to account for potential population stratification.
Population characteristics	The trans-eQTL analysis was based on previously published gene expression and genotype datasets. No new participants were recruited.
Recruitment	The trans-eQTL analysis was based on previously published gene expression and genotype datasets. No new participants were recruited.
Ethics oversight	We used genotype and gene expression data from ALSPAC, TwinsUK, CoLaus, GEUVADIS, MRCA, MRCE, GENCORD, GTEx v8 and CAP studies. For replication, we used data from the MAGE cohort. The RNA sequencing and genotype data from the GEUVADIS and MAGE studies was publicly available as part of the 1000 Genomes project. For the other studies, we applied for access to individual-level data via relevant data access committees (DACs), explaining the aim of our project and the intent to publicly share meta-analysis summary statistics. Informed consent was obtained when research participants joined the ten studies listed above. The use of the CAP data for this project was approved by the National Heart, Lung and Blood Institute DAC. The use of the GTEx data for this project was approved by the National Human Genome Research Institute DAC. The use of the GENCORD data for this project was approved by the GENCORD DAC. The use of the MRCA and MRCE data for this project was approved by the Gabriel Consortium DAC. The use of TwinsUK data for this project was approved by the TwinsUK Resource Executive Committee. The use of the ALSPAC data for this project was approved by the ALSPAC Executive Committee. For the ALSPAC cohort, ethical approval for the study was obtained from the ALSPAC Ethics and Law Committee and the Local Research Ethics Committees. Consent for biological samples has been collected in accordance with the Human Tissue Act (2004). The CoLaus study was approved by the Institutional Ethics Committee of the University of Lausanne. Single-cell RNA-seq samples were sourced ethically, and their research use was in accord with the terms of informed consent under an institutional review board/ethics committee-approved protocol (UK Regional Ethics Committee approval granted to work at Wellcome Sanger Institute, protocol reference number 15/NW/0282; project was approved by the Ethics on Research Committee of the Institute of Neurobiology at Universidad Nacional Autonoma de Mexico (UNAM),

with the approval number 110.H.).

Note that full information on the approval of the study protocol must also be provided in the manuscript.

Field-specific reporting

Please select the one below that is the best fit for your research. If you are not sure, read the appropriate sections before making your selection.

☒ Life sciences ☐ Behavioural & social sciences ☐ Ecological, evolutionary & environmental sciences

For a reference copy of the document with all sections, see nature.com/documents/nr-reporting-summary-flat.pdf

Life sciences study design

All studies must disclose on these points even when the disclosure is negative.

Sample size	We used all available data from previously published gene expression and genotype datasets.
Data exclusions	To avoid potential confounding by population stratification, we exclude the 87 YRI ancestry individuals from the GEUVADIS dataset and 34 diverse ancestry samples from the GTEx dataset.
Replication	Four of the six trans-eQTL detected in our primary meta-analysis also replicated in an independent cohort of 682 individuals, including the USP18 trans-eQTL locus that is the primary focus of the manuscript.
Randomization	Our manuscript is based on meta-analysis of existing LCL eQTL datasets. As such, randomisation was not relevant for this study.
Blinding	We performed re-analysis of existing datasets where blinding was not relevant.

Reporting for specific materials, systems and methods

We require information from authors about some types of materials, experimental systems and methods used in many studies. Here, indicate whether each material, system or method listed is relevant to your study. If you are not sure if a list item applies to your research, read the appropriate section before selecting a response.

Materials & experimental systems

n/a	Involved in the study	
☒	☐	Antibodies
☒	☐	Eukaryotic cell lines
☒	☐	Palaeontology and archaeology
☒	☐	Animals and other organisms
☒	☐	Clinical data
☒	☐	Dual use research of concern
☒	☐	Plants

Methods

n/a	Involved in the study	
☒	☐	ChIP-seq
☒	☐	Flow cytometry
☒	☐	MRI-based neuroimaging

Plants

Seed stocks	*Report on the source of all seed stocks or other plant material used. If applicable, state the seed stock centre and catalogue number. If plant specimens were collected from the field, describe the collection location, date and sampling procedures.*
Novel plant genotypes	*Describe the methods by which all novel plant genotypes were produced. This includes those generated by transgenic approaches, gene editing, chemical/radiation-based mutagenesis and hybridization. For transgenic lines, describe the transformation method, the number of independent lines analyzed and the generation upon which experiments were performed. For gene-edited lines, describe the editor used, the endogenous sequence targeted for editing, the targeting guide RNA sequence (if applicable) and how the editor was applied.*
Authentication	*Describe any authentication procedures for each seed stock used or novel genotype generated. Describe any experiments used to assess the effect of a mutation and, where applicable, how potential secondary effects (e.g. second site T-DNA insertions, mosiacism, off-target gene editing) were examined.*
